# Supplementary material for: Structures of MmpL complexes reveal the assembly and mechanism of this family of transporters
Source: Sci Adv. 2025 Aug 13;11(33):eadx1129. doi: 10.1126/sciadv.adx1129 (PMC12346298; doi:10.1126/sciadv.adx1129)
Supplement: Supplementary file 1 — Supplementary Text Figs. S1 and S2 Tables S1 and S2 [file sciadv.adx1129_sm.pdf]

Science Advances
AAAS

Supplementary Materials for

Structures of MmpL complexes reveal the assembly and mechanism of this family of transporters

Zhemin Zhang *et al.*

Corresponding author: Edward W. Yu, edward.w.yu@case.edu

Sci. Adv. **11**, eadx1129 (2025)
DOI: 10.1126/sciadv.adx1129

This PDF file includes:

Supplementary Text
Figs. S1 and S2
Tables S1 and S2

Supplementary Information

Secondary structural elements of MmpL5

The TMs, α-helices and β-strands of MmpL5 are assigned numerically from the N- to C-termini: TM1 (25-51)), α1 (51-62), α2 (72-84), β1 (91-99), α3 (105-120), β2 (126-128), α4 (138-141), β3 (148-155), α5 (162-176), β4 (184-189), TM2 (191-225), TM3 (228-255), TM4 (262-293), TM5 (297-328), TM6 (332-365), TM7 (a (379-390) and b (392-412)), α6 (420-422), α7 (428-439), α8 (442-445), β5 (448-453), α9 (461-476), β6 (481-484), α10 (499-505), α11 (508-575), α12 (581-589), α13 (599-672), α14 (677-684), α15 (695-699), α16 (701-710), β7 (717-723), α17 (731-748), β8 (757-761), TM8 (763-797), TM9 (800-826), TM10 (837-866), TM11 (868-899) and TM12 (903-935).

Secondary structural elements of MmpS5

The TM and β-strands of MmpS5 are assigned numerically from the N- to C-termini: TM (6-27), β1 (50-58), β2 (63-68), β3 (74-78), β4 (84-90), β5 (98-103), β6 (107-114), β7 (117-127) and β8 (130-134).

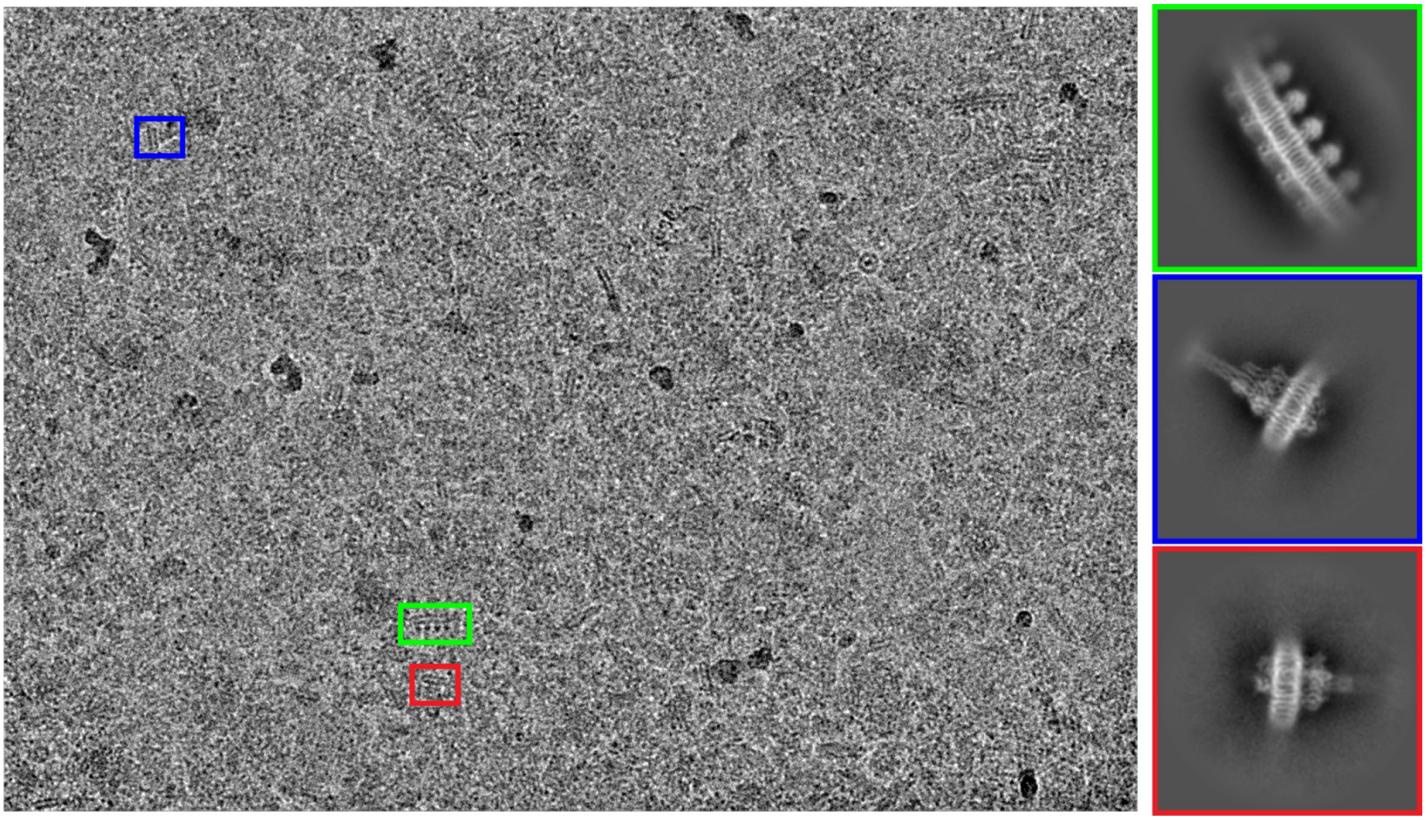

Fig S1. Cryo-EM experimental micrograph. This representative micrograph indicates that the cryo-EM sample contains different protein complexes. The three colored squares highlight single-particle images of three different complexes (green, 2D arrays of the monomeric AcpM-MmpL5 complex; blue, trimeric AcpM-MmpL5-MmpS5 complex; red, trimeric AcpM-MmpL5 complex).

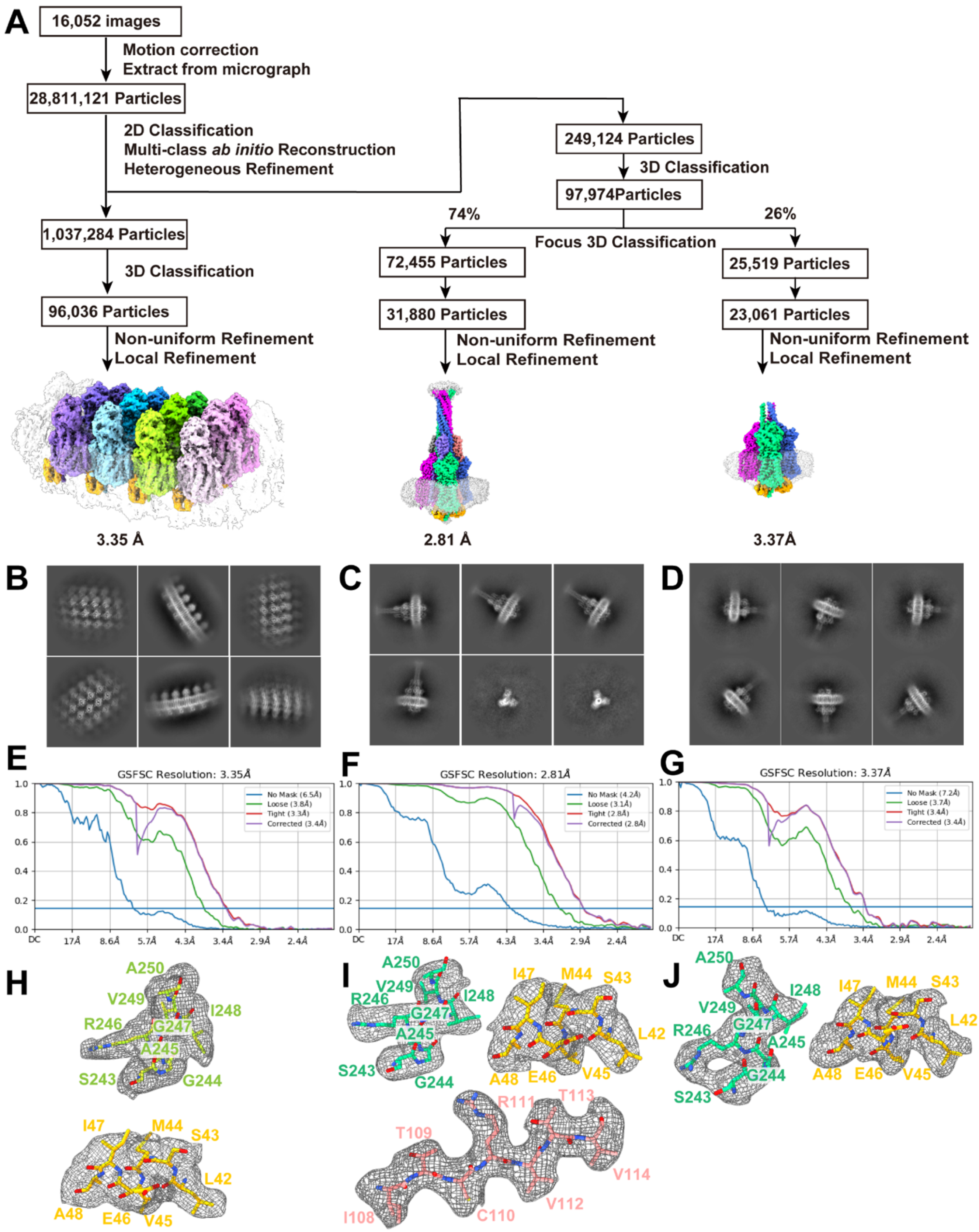

Fig S2. Data processing. (A) Cryo-EM data processing begins as standard workflow, where motion-corrected micrographs are picked, particles undergo 2D classification and initial models are iteratively built. These low-resolution initial models are then used to retrieve particles from the cleaned dataset, resulting in 3 high-resolution maps. These maps lead us to solve cryo-EM structures of the 2D arrays of the monomeric AcpM-MmpL5 complex, the trimeric AcpM-MmpL5-MmpS5 complex and the trimeric AcpM-MmpL5 complex to resolutions of 3.35 Å, 2.81 Å and 3.37 Å, respectively. (B-D) Representative 2D classes of the three complexes. (E-G) Gold-Standard Fourier shell correlation (GS-FSC) curves of the three complexes. (H-J) Representative local density maps of the three complexes.

Table S1. Cryo-EM data collection and refinement statistics.

**Data collection**	**Sucrose cushion sample of *M. Smegmatis* membrane expressed with MmpL5/S5**		
Magnification		81,000	
Voltage (kV)		300	
Electron Microscope		Krios-GIF-K3	
Defocus (um)		-0.8 to -1.5	
Energy filter width (eV)		20	
Pixel size (Å)		1.07 (0.535)	
Total dose (e^-/ Å^2)	40.2	50	50
Number of frames	46	40	40
Number of micrographs	3,855	6,201	5,996
Number of Initial particles		28,811,121	
**Refinement**	**Monomeric AcpM-MmpL5**	**Trimeric AcpM-MmpL5-MmpS5**	**Trimeric AcpM-MmpL5-MmpS5**
Number of total particles	96,036	31,880	23,061
GS-FSC Resolution (0.143, Å)[a]	3.35	2.81	3.37
Model composition			
Chains	32	9	6
Protein residues	9,684	3,468	2,589
Ligand	8	3	3
r.m.s.d.			
Bond lengths (Å)	0.002	0.005	0.003
Bond angles (°)	0.388	0.520	0.475
Reconstruction rotational symmetry	C1	C3	C3
**Validation**			
MolProbity score	1.62	1.51	1.48
Clash score	7.42	4.43	5.27
Ramachandran plot			
Favored (%)	98.94	98.41	98.87
Allowed (%)	1.06	1.59	1.13
Disallowed (%)	0.00	0.00	0.00
CC Mask[b]	0.66	0.81	0.81

[a]Gold-Standard Fourier-Shell Correlation

[b]Correlation Coefficient Mask

Table S2 Proteomic analysis.

Ranking	Protein ID	Accession	Mass kDa	Peptide unique	Seq. Coverage (%)	Sequest Score
1	**MmpL5 protein**	**A0QS80**	**105.4**	**60**	**54.0**	**4290.09**
2	Cytochrome bc1 complex Rieske iron-sulfur subunit	I7GD61	46.3	35	66.0	1328.80
3	ATP-dependent zinc metalloprotease FtsH	I7GG40	83.5	39	62.0	1060.13
4	cytochrome-c oxidase	I7GD63	38.0	28	58.0	925.52
5	Elongation factor Tu	A0QS98	43.7	22	78.0	874.20
6	NADH:ubiquinone reductase (non-electrogenic)	I7GBR7	49.0	22	71.0	840.85
7	ATP synthase subunit beta	A0R200	51.6	30	88.0	834.46
8	UPF0182 protein MSMEG_1959/MSMEI_1915	A0QTT7	109.0	36	53.0	820.69
9	DNA-directed RNA polymerase subunit beta'	A0QS66	146.4	68	67.0	795.19
10	Phage shock protein A, PspA	I7G0I2	30.3	22	90.0	714.75
:						
14	**MmpS5 protein**	**A0QS79**	**14.9**	**8**	**80.0**	**614.55**
:						
280	**Meromycolate extension acyl carrier protein (AcpM)**	**A0R0B3**	**10.7**	**5**	**36.0**	**94.48**
